# The efficacy of heart rate variability biofeedback in patients with acute ischemic stroke: A protocol for systematic review and meta-analysis

**DOI:** 10.1097/MD.0000000000031834

**Published:** 2022-11-18

**Authors:** Lihua Xie, Chunyan Zhang, Junling Zhang, Min Zhao

**Affiliations:** a Department of Neurology, Linfen People’s Hospital, Linfen, Shanxi Province, China; b Department of Internal Medicine, Taiyuan University of Technology Hospital, Taiyuan, Shanxi Province, China.

**Keywords:** heart rate variability biofeedback, ischemic stroke, meta-analysis, protocol

## Abstract

Stroke is the most common serious neurological disorder, and in high-income countries, it is the fourth-leading cause of death, long-term disability, and reduced quality of life among adults. Heart rate variability (HRV) can improve autonomic dysfunction, cognitive impairment, and psychological distress in other patient populations, but its effect in patients with acute ischemic stroke is still unclear. We conducted a protocol for systematic review and meta-analysis to evaluate the efficacy of HRV biofeedback in patients with acute ischemic stroke.

**Methods:**

A computerized literature search will be performed in the following electronic databases from their inceptions to October 2022: PubMed, EMBASE, MEDLINE, Cochrane Central Register of Controlled Clinical Trials, China Knowledge Resource Integrated Database, Wanfang Data Information, and Weipu Database for Chinese Technical Periodicals. The risk of bias in the included articles is assessed according to the Risk of Bias Assessment Tool in Cochrane Handbook of Systematic Reviews. Data are analyzed with the Review Manager Version 5.3 software.

**Results:**

This paper will provide high-quality synthesis to assess the efficacy of HRV biofeedback in patients with acute ischemic stroke.

**Conclusion:**

HRV biofeedback may be a promising intervention for improving autonomic function, cognitive impairment, and psychological distress in patients with acute ischemic stroke.

## 1. Introduction

According to the World Health Organization, stroke is a condition with no apparent cause other than vascular origin with symptoms lasting for more than 24 hours or leading to death with a rapidly developing clinical sign of focal or global disturbances of cerebral function.^[1,2]^ Stroke is the most common serious neurological disorder, and in high-income countries, it is the fourth-leading cause of death, long-term disability, and reduced quality of life among adults.^[3,4]^ According to the American Stroke Association, about 87% of the cases are ischemic, and the remaining 13% are hemorrhagic.^[5,6]^ The most common symptoms include paralysis (in 1 or both sides), loss of balance, and spasticity, which commonly appear days or weeks after the occurrence of a stroke.^[7]^

Heart rate variability (HRV), oscillations of instantaneous heart rates and between heartbeats (R-R intervals), represents 1 of the most promising early and quantitative markers of autonomic dysfunction.^[8,9]^ HRV has been used to detect conditions from fetal distress to diabetic neuropathy in advance of other markers of autonomic dysfunction since the 1960s.^[10]^ Today, HRV measurements are performed with standardized instruments, allowing for more precise clinical correlations among various HRV indices (time- and frequency-domain indices). In poststroke patients, significantly lower HRV indices are an indicator of autonomic dysfunction.

HRV biofeedback is a behavioral intervention that trains subjects to obtain voluntary control of the higher amplitude of HRV.^[11]^ By pacing breathing at a slow frequency so that resonance occurs between cardiac rhythm and respiration, subjects are able to enhance their HRV indices.^[12]^ HRV biofeedback has been associated with beneficial health outcomes. Research conducted over the past 2 decades or more has confirmed the efficacy of HRV biofeedback in improving autonomic, cognitive, and psychological well-being in a variety of disease states.^[13,14]^ However, the effects of HRV biofeedback in patients with acute ischemic stroke have not been explored. Thus, we conducted a protocol for systematic review and meta-analysis to evaluate the efficacy of HRV biofeedback in patients with acute ischemic stroke.

## 2. Methods

This review protocol is registered in the international prospective register of systematic review. The registration number was CRD42022369167. The systematic review will be conduct by Cochrane Handbook for Systematic Reviews of Interventions guidelines and reported according to preferred reporting items for the Systematic Review and Meta-Analysis Protocols guidelines.^[15]^ The ethical approval or informed consent was not required in this study because it belongs to secondary research which based on some previously published data.

### 2.1. Eligibility criteria

#### 2.1.1. Study designs to be included.

We will only include randomized controlled trials (RCTs), non-RCTs, quasi-RCTs, reviews and other types of studies will be excluded.

#### 2.1.2. Participant or population.

This review includes acute ischemic stroke patients regardless of race, region, and sex.

#### 2.1.3. Intervention.

The intervention group received at least 9 sessions of HRV biofeedback over 3 days in addition to comprehensive stroke unit care.

#### 2.1.4. Control.

The control group received sham biofeedback.

#### 2.1.5. Outcomes.

Repeated measures of HRV, mini-mental status examination, and Hospital Anxiety and Depression Scales were collected prior to and at 1 and 3 months postintervention.

### 2.2. Search strategy

A computerized literature search will be performed in the following electronic databases from their inceptions to October 2022: PubMed, EMBASE, MEDLINE, Cochrane Central Register of Controlled Clinical Trials, China Knowledge Resource Integrated Database, Wanfang Data Information, and Weipu Database for Chinese Technical Periodicals. The following key terms will be used in combination to develop search strategy for each electronic database: stroke, HRV and randomized. The literature search strategy is summarized for PubMed in Table 1. The reviewers will screen the reference lists of eligible studies and relevant reviews to identify additional sources of information. Search results will be compiled using the citation management software EndNote X9.

**Table 1:** Search strategy for PubMed.

#1 stroke [Title/Abstract]
#2 cerebral haemorrhage [Title/Abstract]
#3 hematencephalon [Title/Abstract]
#4 encephalorrhagia [Title/Abstract]
#5 cerebrovascular disease [Title/Abstract]
#6 cerebellar haemorrhage [Title/Abstract]
#7 basal ganglia hemorrhage[Title/Abstract]
#8 thalamic haemorrhage [Title/Abstract]
#9 intraventricular haemorrhage [Title/Abstract]
#10 #1 OR # 2 OR # 3 OR #4 OR #5 OR #6 OR #7 OR #8 OR #9
#11 heart rate variability biofeedback [Title/Abstract]
#12 HRV biofeedback [Title/Abstract]
#13 biofeedback device [Title/Abstract]
#14 biofeedback system [Title/Abstract]
#15 #11 OR #12 OR #13 OR #14
#16 #10 AND #15

### 2.3. Selection of eligible studies

**Figure 1.**
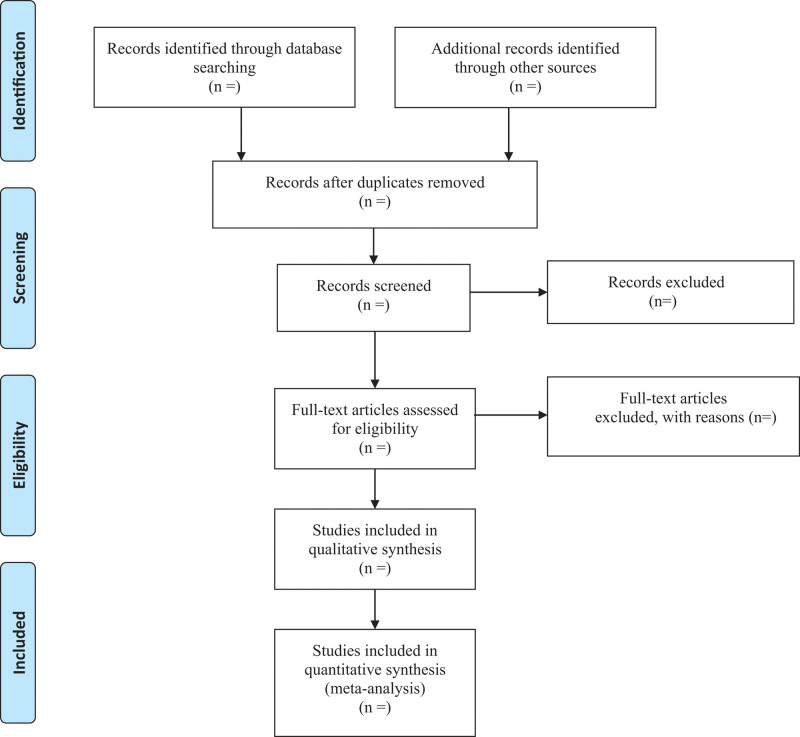
Flowchart of the study screening process.

Two reviewers will independently screen the titles and abstracts of the retrieved articles. We will also acquire the full text for screening to evaluate the eligibility for inclusion when necessary. All articles are published in English or Chinese. Any disagreements will be resolved by discussion among reviewers. The process and results of the studies selection will be presented in a flow chart with Figure 1.

### 2.4. Data collection

Two authors will independently separate the result information from each investigation according to a standardized data extraction form. The disagreement will be settled regarding study inclusion by a third reviewer as necessary. We will use the software to remove the duplicate paper and then use manual de-duplication. A template form will be utilized to collect data. The extracted data items will contain the authors, years of publication, study designs, sample sizes, interventions, primary outcome, other outcomes, adverse effects, and funding source.

### 2.5. The risk of bias in individual studies

Two independent reviewers will separately survey methodological quality utilizing the Cochrane risk of bias tool.^[16]^ The conflicts cannot be settled in the review will search consensus for a third author as required. Domains need to be evaluated will include: sequence generation; allocation concealment; blinding of participants; blinding of outcome assessment; incomplete outcome data; selective outcome reporting; and other issues.

### 2.6. Data synthesis and analysis

If possible, the meta-analysis will be performed with the Review Manager Version 5.3 software (Copenhagen, The Nordic Cochrane Centre, the Cochrane Collaboration, 2014). For the continuous data, the changes from baseline will be used in the meta-analysis. A random effects model will be used for a better analysis of the clinical heterogeneity. If outcome measure scales are the same, the mean difference and 95% confidence intervals will be calculated. In the case of different outcome measure scales, the standardized mean difference and 95% confidence intervals will be calculated. According to the recommendations of the Cochrane handbook for systematic reviews of interventions, the heterogeneity will be assessed using *I*^2^ statistic (where *I*^2^ > 30% indicated moderate heterogeneity; *I*^2^ > 50% substantial heterogeneity; and *I*^2^ > 75% considerable heterogeneity) and Cochran Q statistic (considered to be statistically significant when *P* < .10). The subgroup analysis will be conducted based on different control interventions and subpopulations if there are more than 3 eligible studies. The risk of publication bias will be assessed by funnel-plot if more than 10 trials are included in the meta-analysis. If relevant data are not reported, the original authors will be contacted to request the missing data, especially for those necessary for the meta-analysis. If the meta-analysis is not possible, a narrative synthesis of the available data will be conducted.

## 3. Discussion

Multiple clinical studies have shown that HRV biofeedback can alleviate neurocardiac dysfunction and improve clinical outcomes in neuropsychiatric and cardiovascular disorders, possibly mediated by augmented respiratory sinus arrhythmia triggering increased baroreflex gain and parasympathetic outflow.^[17,18]^ This would explain why in previous controlled studies HRV biofeedback was able to counteract a shift of the autonomic balance toward the sympathetic nervous system and was therefore particularly efficacious in improving cardiac autonomic function in conditions that are associated with chronic sympathetic hyperactivity such as depression, addiction to alcohol and posttraumatic stress disorder. Notably, in these studies the beneficial effects of HRV biofeedback exceeded mere neurocardiac improvement but also translated into alleviation of clinical outcomes such as depressive symptoms and craving for alcohol.^[19,20]^ Currently, few studies have reported HRV biofeedback in stroke. This is the first meta-analysis to evaluate the efficacy of HRV biofeedback in patients with acute ischemic stroke. Some limitations of this study should be noted. Our studies only search database in English and Chinese because of language barriers. So, there may exist a language bias. We will do a full-scale in the future to evaluate it better. Then, the large clinical heterogeneity may exist for different disorder stage and duration of treatment and action consistency, future study will be more noticed those limitations.

## Author contributions

**Conceptualization:** Chunyan Zhang.

**Investigation:** Junling Zhang.

**Writing – original draft:** Lihua Xie.

**Writing – review & editing:** Min Zhao.
